# Utilization of okra *(Abelmoschus esculentus L.) *and its byproducts for developing novel nutrient-rich food products

**DOI:** 10.1038/s41598-026-47671-8

**Published:** 2026-05-07

**Authors:** Eman R. M. Abbas, Tahany A. A. Aly, Mohamed A. Kelany, Emam A. Abdel-Rahim, Ammar AL-Farga, Sara M. Mohamed

**Affiliations:** 1 Food Technology Research Institute, Agriculture Research Center, Ministry of Agriculture, Giza, Egypt; 2 Regional Center for Food and Feed, Agriculture Research Center, Ministry of Agriculture, Giza, 12619 Egypt; 3 Food Science and Nutrition Department, Faculty of Agriculture, Sohag University, Sohag, Egypt; 4 Biochemistry Department, Faculty of Agriculture, Cairo University, Giza, Egypt; 5 Biological sciences, College of science, University of Jeddah, Jeddah, Saudi Arabia

**Keywords:** Okra, Biscuits, Organoleptic properties, Pods, Amino acid profile, Biochemistry, Proteins

## Abstract

Okra (*Abelmoschus esculentus* L.) stands out for its high nutritional value, providing significant bioactive compounds found in its mucilage, seeds, and pods. Rich in vitamins and carbohydrates, okra seeds serve as an excellent protein source, especially due to their high quality and essential amino acid profile when compared to other plant proteins. This study aims to explore the potential of incorporating okra flour and okra waste flour into cracker formulations, creating a high-nutrient snack that offers dietary fiber. Using a standard cracker recipe, variations were developed by substituting 5% and 10% of the flour content with dried fresh okra, dried boiled okra, and dried okra waste (pods and peels). Proximate analysis dry weight based showed similar moisture content (8.5–9.4%), with fresh okra exhibiting the highest protein content (20.6%) and waste okra the highest crude fiber (20.78%). Mineral analysis highlighted fresh okra’s rich calcium, iron, and magnesium content. Sensory evaluations indicated that crackers with 5% okra flour were most acceptable in taste and texture, suggesting the potential of okra to enhance the nutritional profile of snack foods while preserving sensory qualities.

## Introduction

Okra (*Abelmoschus esculentus* L.) is one of the earliest cultivated crops and is now grown in diverse regions, including Africa, Asia, southern Europe, and the Americas. Belonging to the Malvaceae family, okra is classified as a warm-season annual herbaceous vegetable^1^. The Food and Agriculture Organization of the United Nations (FAOSTAT) reports that global okra production reached approximately 10.5 million tons in 2020, with cultivation across 2.53 million hectares. This remarkable vegetable not only serves as a staple in numerous cuisines but also offers a wealth of nutritional benefits^2,3^. Okra is recognized for its impressive health-enhancing properties, which include anti-fatigue, anti-diabetic, antioxidant, and notably, anticancer effects^4^. Its polysaccharides and fibers provide alternative energy sources and biotherapeutics that have significant potential for developing essential medications^5^. Rich in antioxidants, polyphenols, and various phytochemicals, okra plays a crucial role in improving health and the functional properties of foods^6^. The phytoconstituents in okra demonstrate powerful antidiabetic, antihyperlipidemic, neuroprotective, chemoprotective, and radical scavenging properties. Its lipophilic and hydrophilic antioxidant components effectively neutralize free radicals in both lipid and aqueous environments. Moreover, the flavonoids and vitamin C found in okra work together synergistically to protect low-density lipoproteins from oxidation^7,8^. Research indicates that extracts from okra’s peel and seeds display antihyperlipidemic and antidiabetic effects in rats with streptozotocin-induced diabetes^8,9^. Additionally, a study revealed that incorporating 20% okra seed flour into noodle dishes significantly reduced insulin and glucose levels in healthy, non-diabetic individuals^10^. Regular consumption of okra ideally at least three times a week can help prevent diabetes and mitigate symptoms of diabetic-induced hyperglycemia^11^. Although the entire okra plant is edible, the young pods are especially valued for their unique flavor and culinary flexibility. They can be consumed in various forms fresh, dried, boiled, or fried and are often used to enhance soups, salads, and stews^12^. In addition, the rich content of biopolymers and bioactive compounds in okra fruits has led to their increasing use in the food and pharmaceutical industries such as emulsifiers, blood volume expanders, and in the production of medicinal tablets. This underscores okra’s significance not only as a nutritious food source but also as a key ingredient in health-related applications^2^. This study aims to utilize okra pods and waste materials to improve the organoleptic properties of crackers with various formulations.

## Materials and methods

### Materials

**Fig. 1:**
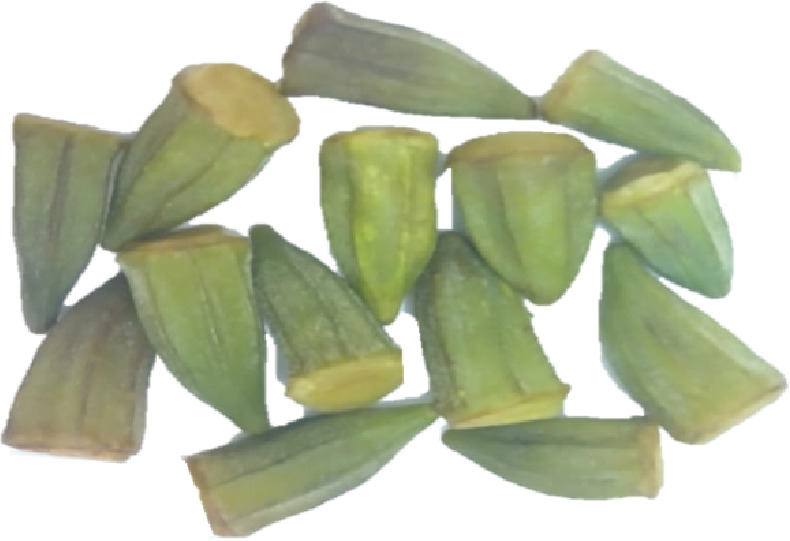
Fresh Okra (*Abelmoschus esculentus* L.) Manshaa variety from local market.

After purchasing fresh okra (Fig. 1) from the local market, the fruits were thoroughly cleaned, and the tops were discarded as waste. The okra was then dried for two days at 50 °C. Three okra powder fractions were prepared: fresh okra powder, boiled okra powder, and okra waste powder, each obtained by grinding the respective material and passing it through a 1.2 mm screen sieve using a heavy-duty grinder. The powdered okra was stored in glass containers at 4 °C in the refrigerator until it was ready for chemical analysis.

### Preparation of okra cracker

**Fig. 2:**
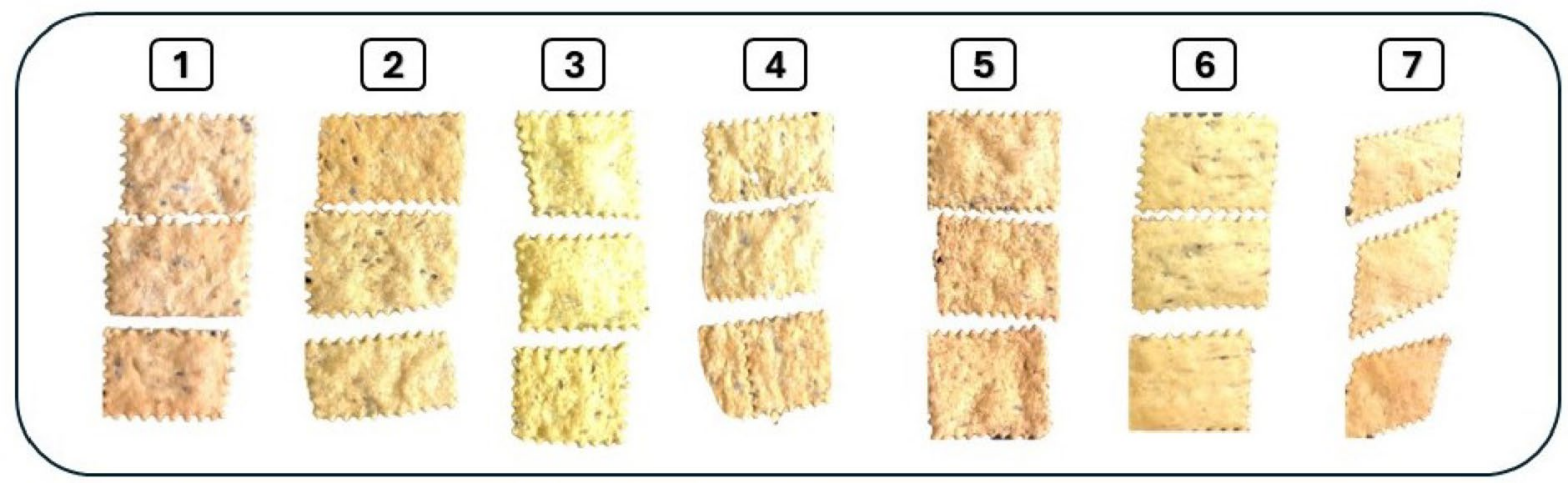
Produced crackers from okra and okra waste; (1) Control without okra, (2) 5% okra fresh powder, (3) 10% okra fresh powder, (4) 5% okra boiled powder, (5) 10% okra boiled powder, (6) 5% okra waste powder and (7) 10% okra waste powder.

The preparation of okra crackers was conducted following the method outlined by^13^. The basic formulation for the cracker biscuits consisted of 100 g of flour, 6 g of fat, 2 g of salt, 4 g of sugar, 2 g of baking powder, and 30 g of water. Initially, wheat flour, 5 g and 10 g (5% and 10%) of okra flour (prepared from cleaned, airdried and grounded with laboratory blender), and other ingredients were combined, and water was gradually added to form a dough. The dough was then rolled out to a thickness of 3 mm. Using a cutter, the dough was shaped into crackers, which were baked at 200 °C for 10 to 15 min. After baking, the crackers were cooled to ambient temperature and packaged in high-density polyethylene bags. Different formulations were created: one served as a control (C) using only wheat flour without okra, while the others incorporated varying percentages of dried fresh okra, dried boiled okra, and dried okra waste, specifically at 5% and 10% substitutions for the wheat flour as presented in Fig. 2.

### Chemical analysis

#### Proximate determination

Moisture, ash, protein, crude fiber, and fat content of okra and okra fractions was carried out according to the methods of AOAC^14^. Non-nitrogen extract or total carbohydrate was calculated by difference as shown in the following equation.

$${\rm{Total Carbohydrates \% = 100 - (moisture + ash + protein + fat + fiber)}}.$$

#### Determination of elements content

Okra’s mineral contents were calculated in accordance with AOAC^14^. Using a Perkin-Model 3300 atomic absorption spectrophotometer (USA).

#### Determination of vitamins

The^15^ method was used to assess vitamin C (ascorbic acid), by blending a known sample mass with oxalic acid–EDTA, then filtering and centrifuging. A measured aliquot of the extract (or juice plus reagent) is treated with color-developing reagents, left 15 min, and the blue solution’s absorbance at 760 nm is compared with a calibration graph. while^16^ was used to determine vitamin E (tocopherol) and vitamin A (β-carotene).

#### Determination of fatty acid

The fatty acid composition was analyzed using gas chromatography (GC) following the method described by^17^. Fatty acid methyl esters were separated using an Agilent 6890 series GC apparatus equipped with a DB-23 column (60 m × 0.32 mm × 0.25 μm). The oven temperature was initially set to 150 °C and then ramped to 195 °C at a rate of 5 °C/min, followed by an increase to 220 °C. The flow rate during the analysis was maintained at 1.5 ml/min.

#### Determination of amino acids

Sample aliquots (okra or okra by products) containing approximately 8–12 mg of protein was placed in a 20mL cuvette and combined with 9 mL of 6 M HCl^18^. After sealing the cuvette, the samples were hydrolyzed at 110 °C for 24 h under a nitrogen atmosphere. The resulting hydrolysates were then transferred to a 100 mL volumetric flask, mixed with 9 mL of 6 M NaOH, and diluted with 0.02 N HCl. Subsequently, all samples were filtered and analyzed using a Hitachi L-8800 amino acid analyzer (Tokyo, Japan).

### Phytochemicals analysis

#### Determination of total phenolics and flavonoids contents

The total amounts of flavonoids and phenols were measured using the Folin-Ciocalteu method and the aluminum trichloride (AlCl_3_) technique, as described by^19^. Total polyphenols content (TPC) was assessed at a wavelength of 750 nm, while total flavonoids content (TFC) was measured at 510 nm using a UV-V spectrophotometer (Jenway 6800, UK). Gallic acid and quercetin were used as standards for TPC and TFC assays, respectively, with quantification based on standard curves for quercetin (10–100 mg/L) and gallic acid (10–100 mg/L).

#### Antioxidant capacity

The total antioxidant capacity of the samples was determined by^20^ methods. A 0.5 ml aliquot of sample methanol extract (0.02 g/ml) was mixed with a 4.5 ml reagent solution (DPPH) and incubated in a boiling water bath. The absorbance of each sample was then measured at 695 nm against a blank using a UV-2450 spectrophotometer.

#### Phytochemical’s determination

The phytochemical components were analyzed using the GC-MS methodology as outlined by^21^. The analysis was performed with an Agilent 7000 Triple Quad mass selective detector (MSD) and an Agilent HP-5ms capillary column, coupled with an Agilent Technology 7890 A gas chromatograph. Identification of the components was achieved by comparing the mass spectra of the analyzed compounds with those known substances, utilizing computer matching with the NIST library and comparing the fragmentation patterns with documented data in the literature.

#### Sensory evaluation

Twelve trained taste panel members assessed the sensory qualities of various cracker treatments, including color, taste, crispness, aroma, and overall acceptability^22^. On the evaluation day, each cracker sample was assigned a random code. Panelists rinsed their mouths with distilled water between tastings. After receiving the samples in a random order, judges were instructed to assign scores for each parameter to reflect their level of acceptance as a score from 1 to 10 points.

### Statistical analysis

All analyses were carried out at triple. The obtained data was reported as mean ± standard deviation. Then compared to the independent samples by T-test. The one-way variance has been applied at a 95% confidence interval with the SPSS 20.0 package program (SPSS Inc., Chicago, USA).

## Results and discussion

### Proximate determination

**Fig. 3:**
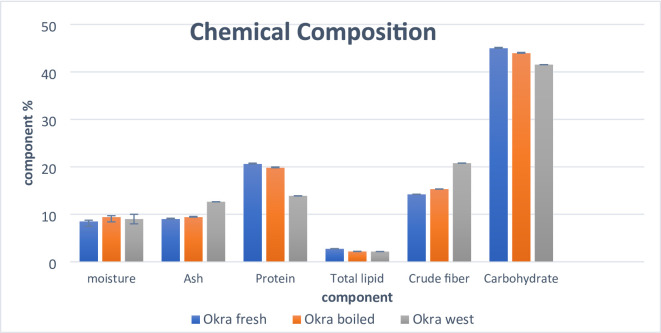
Chemical Composition of fresh, boiled and okra waste powder as % on dry weight.

The proximate composition of fresh, boiled, and waste okra powder is summarized in Fig. 3. The moisture content for fresh, boiled, and waste okra powder ranged from 8.5 to 9.4% and showed no significant differences among the samples. The ash content, which reflects the total mineral quantity present, varied from 9.0 to 12.65% on a dry weight basis in all samples, indicating that okra can provide essential minerals necessary for body development, consistent with findings by^23^. The protein content between fresh and boiled okra powder was comparable, measuring 20.6% and 19.8%, respectively, while the okra waste had the lowest protein content at 13.9%. These findings align with reports by^10,24^. Furthermore, the fat content across fresh, boiled, and waste okra powder showed no significant differences. The highest crude fiber content was found in okra waste, at 20.78%.

### Element’s content of fresh, boiled and okra waste

**Fig. 4:**
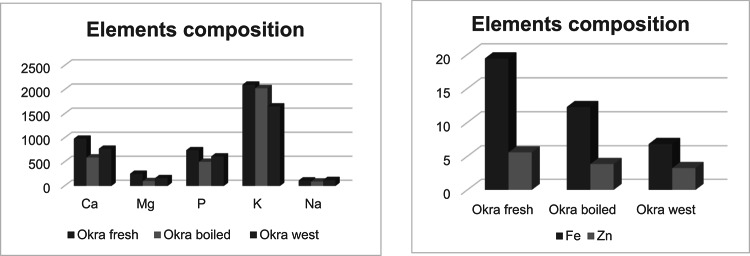
Element’s composition of fresh, boiled and okra waste powder as ppm.

Minerals play a vital role in human nutrition by aiding in the maintenance of acid-base balance, facilitating nerve responses to physiological stimulation, and supporting blood clotting^24^. The data presented in Fig. 4 shows the concentrations of several mineral elements measured in ppm for fresh, boiled, and waste okra powder, specifically calcium (Ca), iron (Fe), magnesium (Mg), phosphorus (P), potassium (K), sodium (Na), and zinc (Zn). The results indicated that fresh okra contained higher levels of Ca, Fe, Mg, P, K, and Zn, with concentrations of 980, 19.4, 250.57, 740.7, 2100, and 5.58 ppm, respectively, compared to the boiled and waste okra. Conversely, okra waste had a higher sodium content at 120.34 ppm^25^. reported that raw okra contains 6.2 ppm of Fe, 5.8 ppm of Zn, 570 ppm of Mg, 2990 ppm of K, and 820 ppm of Ca. This trend and similar values have been documented by other researchers in different types of okra^24,26^.

### Vitamins of okra

The vitamins are a disparate group of compounds; they have little in common either chemically or in their metabolic functions. Nutritionally, they form a cohesive group of organic compounds that are required in the diet in small amounts (micrograms or milligrams per day) for the maintenance of normal health and metabolic integrity^27^. Data in Table 1 show that vitamins present in fresh okra, boiled okra and okra waste. Vitamin A contents 1104.4 ,1015.3 and 1019.6 mg/kg, respectively. The results showed that fresh okra recorded high significant amounts (10.7 and 8.47 mg /kg., respectively) of ascorbic acid and α tocopherol content than boiled okra and okra waste. These results are close to those reported by^9,12,27^. Vitamin C enhances absorption of inorganic iron and inhibits the formation of nitrosamines in the stomach.

**Table 1:** Vitamins composition of okra (fresh, boiled and waste) mg/kg.

Samples	Antioxidative vitamins (mg/kg) dry weight		
	(β carotene) Vitamin A	(Ascorbic acid) Vitamin C	(αTocopherol) Vitamin E
Okra fresh	1104.4 ± 30.9^a^	10.70 ± 0.42^a^	8.47 ± 0.38^a^
Okra boiled	1015.30 ± 91^a^	8.08 ± 0.53^b^	6.20 ± 0.31^b^
Okra waste	1019.6 ± 28.54^a^	0.03 ± 0.003^c^	0.023 ± 0.002^c^

All values are represented as mean ± S.D.

Means with different letters are significantly different at (*p* < 0.05).

Table 1 presents a detailed overview of the vitamin composition of okra in three forms: fresh, boiled, and waste, measured in mg/kg on a dry weight basis. This comparison is essential for understanding okra’s nutritional profile and the effects of cooking methods and waste utilization on its vitamin content. Fresh okra boasts the highest concentration of β-carotene at 1104.4 mg/kg, which serves as a precursor to Vitamin A, vital for healthy vision and immune function. However, boiling reduces the β-carotene level to 1015.30 mg/kg due to nutrient leaching into water and heat-induced degradation of carotenoids. Notably, okra waste retains a β-carotene concentration of 1019.6 mg/kg, which is close to that of boiled okra, though still lower than that of fresh okra. This indicates that the discarded parts of the okra plant still have valuable antioxidants, suggesting potential uses such as animal feed or compost. Regarding Vitamin A (ascorbic acid), fresh okra contains 10.70 mg/kg, essential for immune function, skin health, and vision. Boiling reduces the Vitamin A content to 8.08 mg/kg, as heat-sensitive vitamins can degrade during cooking, leading to the loss of soluble vitamins. The Vitamin A concentration in okra waste is significantly lower at 0.03 mg/kg, highlighting that while the edible parts are rich in this vitamin, the waste is not a viable source, reinforcing the need to consume the fresh portions for better nutrient intake.

Fresh okra also provides 8.47 mg/kg of Vitamin C, an important antioxidant that supports collagen formation and immune health. Upon boiling, the Vitamin C level decreases to 6.20 mg/kg due to its heat sensitivity. The waste from okra shows an extremely low Vitamin C content of 0.023 mg/kg, further underscoring those substantial nutritional benefits primarily come from fresh edible parts. Additionally, fresh okra has a significant Vitamin E content of 8.47 mg/kg, which is crucial for cellular function and acts as a powerful antioxidant. Like the other vitamins, boiling lowers the Vitamin E concentration to 6.20 mg/kg, with waste displaying negligible levels of this vitamin, confirming that these nutrients are mainly found in the edible portions of the plant.

These findings align with those of^12,27,28^. Vitamin C enhances the absorption of inorganic iron and inhibits the formation of nitrosamines in the stomach.

### Fatty acids of okra

The fatty acid composition serves as a critical indicator of the nutritional value of oils. Table 2 presents the concentration percentages of fatty acids in fresh, boiled, and waste okra powder. Understanding the fatty acid composition of okra is essential for evaluating its nutritional value and the impact of cooking on its profile. Myristic acid (C14:0) decreases from 0.56 mg in fresh okra to 0.41 mg when boiled and is not present in waste. Palmitic acid (C16:0), the predominant saturated fatty acid, reduces from 22.63 mg in fresh okra to 20.97 mg when boiled but increases to 26.45 mg in waste, suggesting a concentration effect from water loss during cooking. Monounsaturated fatty acids (MUFAs), such as oleic acid (C18:1n9), decline from 9.66 mg in fresh to 7.86 mg when boiled, and further to 6.56 mg in waste, indicating some nutrient loss. In contrast, polyunsaturated fatty acids (PUFAs), particularly linoleic acid (C18:2n6), increase from 41.45 mg in fresh to 46.49 mg in waste, highlighting the nutritional benefits of all okra parts. Cooking methods deserve further research to optimize nutrient retention in dietary recommendations. Overall, okra contains relatively low lipid levels, with minimal variation among the samples: fresh okra at 2.72%, boiled okra at 2.16%, and okra waste at 2.14%. Linoleic acid is the predominant fatty acid, ranging from 41.45 to 46.49%, followed by palmitic acid, which varies between 20.37% and 26.45%, being most concentrated in okra waste. Other notable fatty acids include linolenic acid (12.15–13.88%), oleic acid (6.56%–9.66%), and stearic acid (2.68–3.37%), along with trace amounts of additional fatty acids. Linoleic acid, an essential omega-6 fatty acid, plays an important role in the biosynthesis of arachidonic acid and certain prostaglandins^18^. Variations in fatty acid composition may result from the transformation of existing fatty acids or the introduction of new fat-soluble compounds during cooking^29^. identified 39 lipid compounds in okra fruits, encompassing classes such as oxylipins, phospholipids, glycolipids, and sphingolipids. Existing literature confirms that okra is rich in various fatty acids, predominantly linoleic, palmitic, oleic, and stearic acids^18,24,30^.

**Table 2:** Fatty acid profile (%) of fresh, boiled, and wastes of okra fruits.

Fatty acids		Okra fresh	Okra boiled	Okra waste (FAO/WHO (2013)
C14:0	Myristic acid	0.56	0.41	–
C14:1n5	Tetradecanoic acid	–	0.45	–
C14:1n9		–	0.49	–
C15:0	Pentadecanoic acid	0.27	0.20	–
C15:1n6	10-Pentadecanoic acid	–	0.51	–
C16:0	Palmitic acid	22.63	20.97	26.45
C16:1n7	Palmitoleic acid	2.04	2.43	–
C17:0	Heptadecanoic acid	1.58	1.12	–
C18:0	Stearic acid	3.28	2.68	3.37
C18:1n9	Oleic acid	9.66	7.86	6.56
C18:1n7	Vaccinic acid	0.90	0.70	–
C18:2n6	Linoleic acid	41.45	44.68	46.49
C18:3n4		0.67	0.70	–
C18:3n3	Linolenic acid	12.15	13.88	13.68
C20:0	Arachidic acid	1.08	0.71	1.45
C20:1n9	Gondoic acid	0.34	0.15	–
C20:2n6	Eicosadienoic acid	–	0.16	–
C21:0	Heneicosaenoic acid	0.22	–	–
C22:0	Behenic acid	1.23	0.78	1.99
C22:1n11	Docosaenoic acid	0.62	0.23	–
C22:1n9	Erucic acid	0.32	–	–
C22:2n6	Docosadienoic acid	0.55	0.42	–

### Amino acids of okra

Okra’s nutritional value and amino acid content are shown in Table (3). Glutamic acid (GLU) was the most prevalent non-essential amino acid in the examined samples, with the high concentrations recorded in boiled okra (13.59 g/100 g), fresh okra (11.50 g/100 g), and waste okra (9.64 g/100 g). The next amino acid found in the sample under study was L-aspartic acid, which was present in fresh okra (14.81 g/100 g), boiled okra (7.58 g/100 g), and waste okra (14.24 g/100 g) in that order. Amino acids are the main forms for long-distance translocation of nitrogen inside the shoot and are crucial for the transport of nitrogen from root to shoot as well as numerous other physiological processes in plants^31^. Leucine was a highly abundant essential amino acid in the okra plant, accounting for 5.40 g/100 g of boiled okra, 4.82 g/100 g of waste okra, and 4.66 g/100 g of fresh okra. L-isoleucine was another amino acid; its contents for fresh, boiled, and waste okra were 3.18 g/100 g, 2.95 g/100 g, and 2.86 g/100 g, respectively. The branched-chain amino acid leucine is crucial for regulating cell metabolism and protein synthesis, but it can also boost insulin production in the pancreatic β-cells and serve as an energy source for metabolic processes^32^. Furthermore, L-phenylalanine contents of 3.99 g/100 g in boiled okra, 3.02 g/100 g in waste okra, and 2.77 g/100 g in fresh okra were observed. As an aromatic amino acid, phenylalanine can promote glucan release, thereby influence carbohydrate metabolism and potentially help to reduce insulin resistance^33^. The boiled okra had the highest essential amino acid index (EAAI) value (72.22 g/100 g), followed by the okra waste (70.59 g/100 g) and the fresh okra (66.32 g/100 g). The corresponding biological value (BV) was 67.02 g/100 g, 65.24, and 60.59 for the boiled, waste, and fresh okra, respectively. Generally, to evaluate its protein quality, okra’s amino acid profile was compared to FAO/WHO (2013) standards. While okra is a rich source of lysine, exceeding the quality of many cereal grains, it remains slightly deficient in methionine. Consequently, okra is classified as a high-quality complementary protein that effectively balances nutrient intake in plant-based diets.

**Table 3:** Amino acid composition (%) of fresh, boiled, and wastes of okra fruits.

Amino acid	Okra			Standard casein
Essential AA	Okra fresh	Okra boiled	Okra waste	
Threonine (THR)	2.77	3.08	3.02	**3.78**
Valine (VAL)	3.98	3.79	3.88	**5.82**
Isoleucine (ILE)	2.86	3.18	2.95	**4.54**
Leucine (LEU)	4.66	5.40	4.82	**8.28**
Phenylalanine (PHE)	2.77	3.99	3.02	**4.55**
Histidine (HIS)	2.38	1.87	2.95	**2.55**
Lysine (LYS)	4.81	5.35	5.04	**7.09**
Methionine (Meth)	1.41	1.62	1.44	**2.57**
TEAA	**25.63**	**28.28**	**27.12**	**39.28**
Non-Essential AA				
Aspartic acid (ASP)	14.81	7.58	14.24	**6.32**
Serine (SER)	2.72	2.83	3.17	**4.96**
Glutamic acid (GLU)	11.50	13.59	9.64	**19.90**
Glycine (GLY)	3.20	3.48	3.31	**1.66**
Alanine (ALA)	3.74	4.44	3.53	**2.65**
Tyrosine (TYR)	1.17	2.27	0.58	**5.08**
Arginine (ARG)	5.05	3.84	4.75	**3.29**
Cysteine (CYS)	1.89	1.77	1.08	**0.36**
TNEAA	**44.08**	**39.80**	**40.29**	**54.02**
TARAA(Phe + TYR)	3.93	6.26	3.60	**-**
TSAA(Meth + CYS)	3.30	3.38	2.52	-
TAAA(ASP + GLU)	26.31	21.16	23.88	-
TBAA (ARG + LYS)	9.85	9.19	9.78	-
EAAI%	66.32	72.22	70.59	-
PER	1.53	1.75	1.66	-
BV%	60.59	67.02	65.24	-
Nutritional index	13.13	14.88	9.81	-

EAAI, Essential amino acid index; BV, biological value; PER, The value of protein efficiency ratio; TARAA, Total aromatic amino acids; TSAA, the total Sulphur amino acid; TAAA, Total acid amino acids; TBAA, Total base amino acids.

### Phytochemical compounds of okra

Phytochemical compounds are widely distributed in plants which have gained much attention, due to their antioxidant activities and free radical scavenging abilities which potentially have beneficial implications for human health^34^. The identification and quantification of the phytochemical compounds present in fresh, boiled and waste okra are shown in Table 4. The main of phytochemical compounds in fresh okra were hexadecanoic acid, phytol, isolongifolol and eicosatrienoic acid, some compounds were not detected in fresh okra. α-Tocopherol acetate was detected in fresh okra but not detected in boiled and waste okra. In boiled okra were found erucic acid, batilol, nonadecane and geranyl isovalerate more than which found in fresh and waste okra. There were many phytochemical compounds detected only in waste okra as reported by^35^.

**Table 4:** Phytochemical compounds of fresh, boiled, and wastes of okra fruits.

No	*R*. T	compound	Okra fresh	Okra boiled	Okra waste
1	9	Anethole	2.45	3.44	4.04
2	14	Dodecanoic acid	1.7	0.65	1.13
3	14.2	trans-Farnesol	0.64	0.43	0.51
4	15.1	Tridecanoic acid, 4,8,12-trimethyl-, methyl ester	0.74	2.12	5.86
5	15.5	Erucic acid	2.28	4.94	2.56
6	15.7	Hexadecanoic acid, ethyl ester	31.47	20.01	17.21
7	16.6	Phytol	16.28	14.51	7.62
8	17	Isolongifolol	17.74	14.41	11.06
9	17.1	7,10,13-Eicosatrienoic acid, methyl ester	9.01	6.71	5.99
10	17.2	Octadecanoic acid, ethyl ester	4.73	3.38	2.78
11	18.4	Batilol	3.07	5.06	2.07
12	18.6	Isocalamenediol	1.97	1.05	0.35
13	19.3	Nonadecane	1.1	2.92	0.65
14	19.7	1-Triacontanol	1.27	2.13	0
15	19.9	Ascorbyl Palmitate	2.06	2.44	2.98
16	20.5	(±)-α-Tocopherol acetate	1.36	0	0
17	20.7	Geranyl isovalerate	2.14	10.81	1.79
18	11.1	Geranyl isovalerate	0	0.39	0.6
19	11.4	Digitoxin	0	0.35	0.6
20	11.7	Nonacosane	0	0.39	0.54
21	12.2	Tricosanoic acid	0	0.28	0
22	14.6	Eicosanoic acid	0	0.52	0
23	16.3	Oleic Acid	0	1.37	0
24	16.5	8-Hexadecyne	0	0.58	0
25	17.3	Thunbergol	0	1.08	1.26
26	14.65	Eicosanoic acid	0	0	1.02
27	15.41	Docosanoic acid	0	0	0.49
28	15.98	Biotin	0	0	0.36
29	16.44	1-Octadecene	0	0	0.81
30	16.54	8-Hexadecyne	0	0	6.39
31	16.75	Heptacosanoic acid, methyl ester	0	0	0.86
32	17.44	β-Amyrone	0	0	0.3
33	17.64	Heptadecane	0	0	1.87
34	17.92	Lanosterol	0	0	0.39
35	18.02	β Carotene	0	0	1.25
36	18.21	Vitexin	0	0	0.58
37	19.12	Desmosterol	0	0	0.86
38	19.24	Stanolone	0	0	0.95
39	19.52	Prednisone	0	0	4.69
40	19.75	Lupeol	0	0	1.65
41	20.39	Campesterol	0	0	6.85
42	20.82	Desmosterol	0	0	1.08

### Bioactive compounds of okra

**Fig. 5:**
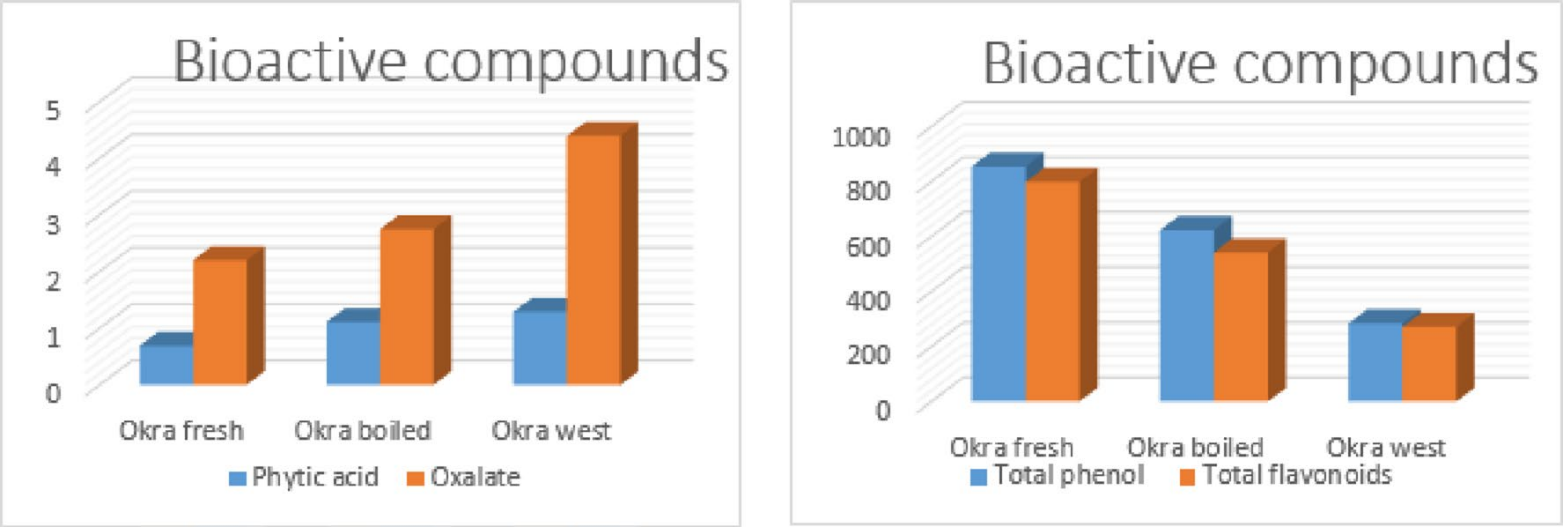
Bioactive compounds of okra (fresh, boiled and okra waste).

Phenolic compounds, which are prevalent in plants, play a crucial role in the human diet and are highly valued for their antioxidant properties. These compounds feature an aromatic ring with one or more hydroxyl groups. Flavonoids, characterized by the C6–C3–C6 structure, make up more than half of the over eight thousand identified phenolic compounds, contributing to their reducing power and free radical scavenging ability^36^. Figure 5 illustrates the content of bioactive compounds in fresh okra, boiled okra, and okra waste. The findings reveal that fresh okra contains significantly higher levels of total phenolics and total flavonoids, measuring 854.6 and 798 ppm, respectively, compared to boiled okra and okra waste. Conversely, okra waste was found to have higher concentrations of phytic acid and oxalate, recorded at 1.29% and 4.40 mg/100 g, respectively. These results are consistent with those reported by^37^.

### Sensory evaluations of crackers samples supplemented with fresh, boiled and waste okra powder

**Fig. 6:**
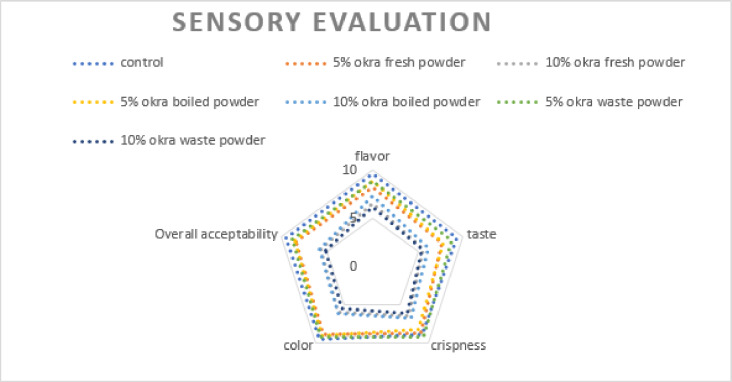
Sensory evaluation of prepared crackers.

Figure 6 illustrates the sensory properties—flavor, taste, crispness, color, and overall acceptability of crackers made with varying concentrations of fresh okra, boiled okra, and okra waste powder. The control sample achieved the highest level of acceptance among panelists. Crackers formulated with 5% of fresh, boiled, and okra waste powder were preferred over those with higher concentrations. In contrast, increasing the okra content to 10% negatively impacted color, flavor, taste, and overall acceptability. The reduced acceptance of the 10% formulations suggests a certain rejection of the strong residual flavor of okra present in these samples. In contrast, the 5% formulations maintained a flavor profile similar to the standard crackers, indicating that this level of okra flour does not significantly alter the flavor. The 10% formulations were also less favored in terms of texture, likely due to the presence of dehydrated okra mucilage, which created an undesirable texture and a sticky consistency, compounded by the high fiber content that reduced extensibility as reported by^38^.

### Effect of various levels of fresh, boiled and waste okra powder on the composition of crackers

The composition of crackers was analyzed following the incorporation of varying levels of fresh, boiled, and waste okra powder, with results presented in Table 5. The addition of okra powder influenced the composition of the cracker biscuits, leading to an increase in moisture, ash, fat, crude fiber, and protein, while total carbohydrate levels decreased. Crackers are among the most widely consumed baked goods globally, and the introduction of novel ingredients into traditional recipes is emerging as a promising strategy to create healthier, more nutritious products in the food industry. Previous studies have shown that incorporating fiber-rich ingredients can enhance the nutritional quality of biscuits^39^. In this study, the control crackers contained 0.72% crude fiber, while those with 5% fresh, boiled, and waste okra powder had crude fiber levels of 1.39%, 1.41%, and 1.74%, respectively. Crackers with 10% fresh, boiled, and waste okra powder contained 2.08%, 2.2%, and 2.77% crude fiber, respectively. This indicates a clear correlation between the amount of incorporated okra powder and the increase in crude fiber, highlighting okra’s potential as a valuable source of dietary fiber, phenolic compounds, flavonoids, and natural antioxidants for future product development. The fiber content rose in proportion to the amount of okra flour added due to okra’s high fiber content^38^. Proteins, as one of the three main macronutrients essential for energy metabolism, were found in higher concentrations in formulations containing okra flour, reflecting okra’s notable protein content. However, the study found no significant difference in lipid content between the okra formulations and the control crackers, likely because okra is low in fat. The observed decrease in carbohydrate content alongside the increased supplementation of okra powder may be attributed to the low carbohydrate levels in okra itself. These findings align with results reported by^38^.

**Table 5:** Chemical composition of prepared crackers with fresh, boiled, and wastes of okra fruits additives.

Samples	Moisture	Ash	Protein	Fat	Fibers	Carbohydrates
Control	5.93 ± 0.13^a^	2.02 ± 0.12^b^	9.34 ± 0.07^b^	12.05 ± 0.13^a^	0.72 ± 0.04^d^	69.94
5% fresh okra	6.13 ± 0.05^a^	2.37 ± 0.06^b^	9.90 ± 0.03^a^	11.55 ± 0.05^a^	1.39 ± 0.04^c^	68.66
10% fresh okra	6.25 ± 0.04^a^	2.77 ± 0.03^ab^	10.49 ± 0.04^a^	11.09 ± 0.08^b^	2.08 ± 0.04^b^	67.32
5% boiled okra	6.17 ± 0.03^a^	2.41 ± 0.03^b^	9.87 ± 0.03^a^	11.49 ± 0.09^a^	1.41 ± 0.04^c^	68.65
10% boiled okra	6.33 ± 0.03^a^	2.80 ± 0.02^ab^	10.37 ± 0.04^a^	11.08 ± 0.03^b^	2.20 ± 0.02^ab^	67.22
5% okra waste	6.16 ± 0.03^a^	2.60 ± 0.03^ab^	9.52 ± 0.03^ab^	11.51 ± 0.03^a^	1.74 ± 0.05^c^	68.47
10% okra waste	6.34 ± 0.05^a^	3.19 ± 0.08^a^	9.8 ± 0.07^a^	11.08 ± 0.03^b^	2.77 ± 0.04^a^	66.82

All values are represented as mean ± S.D. Means with different letters are significantly different at (*p* < 0.05).

## Conclusions

Okra is a valuable functional food, rich in nutrients and bioactive compounds, and can be used to improve the nutritional content of food products. Incorporating okra flour into crackers, for instance, significantly increases their protein and fiber, making them a healthier snack option. Fresh okra offers superior levels of vitamins, minerals, and amino acids compared to boiled or waste okra. While a 5% inclusion of okra powder in crackers results in acceptable flavor and texture, higher concentrations can negatively impact sensory attributes due to strong residual flavors and the texture of okra mucilage. The study emphasizes the potential of okra and its byproducts to enhance dietary fiber, protein, essential fatty acids, and bioactive compounds in snacks, contributing to sustainable food production and innovative ingredient formulations.

## Data Availability

All data generated or analyzed during this study are included in this published article.
